# Supplementary material for: Tead1a Initiates Transcriptional Priming Through the TEAD1a/YAP‐Notch1‐Spi1/Cebpα Axis to Promote Neutrophil Fate
Source: Adv Sci (Weinh). 2025 Aug 28;12(41):e05441. doi: 10.1002/advs.202505441 (PMC12591127; doi:10.1002/advs.202505441)
Supplement: Supplementary file 4 — Supplementary Dataset [file ADVS-12-e05441-s004.zip › 3-2.zip!/3-2/02-2.cluster/cluster4/Enrich/ego_CC.pdf]

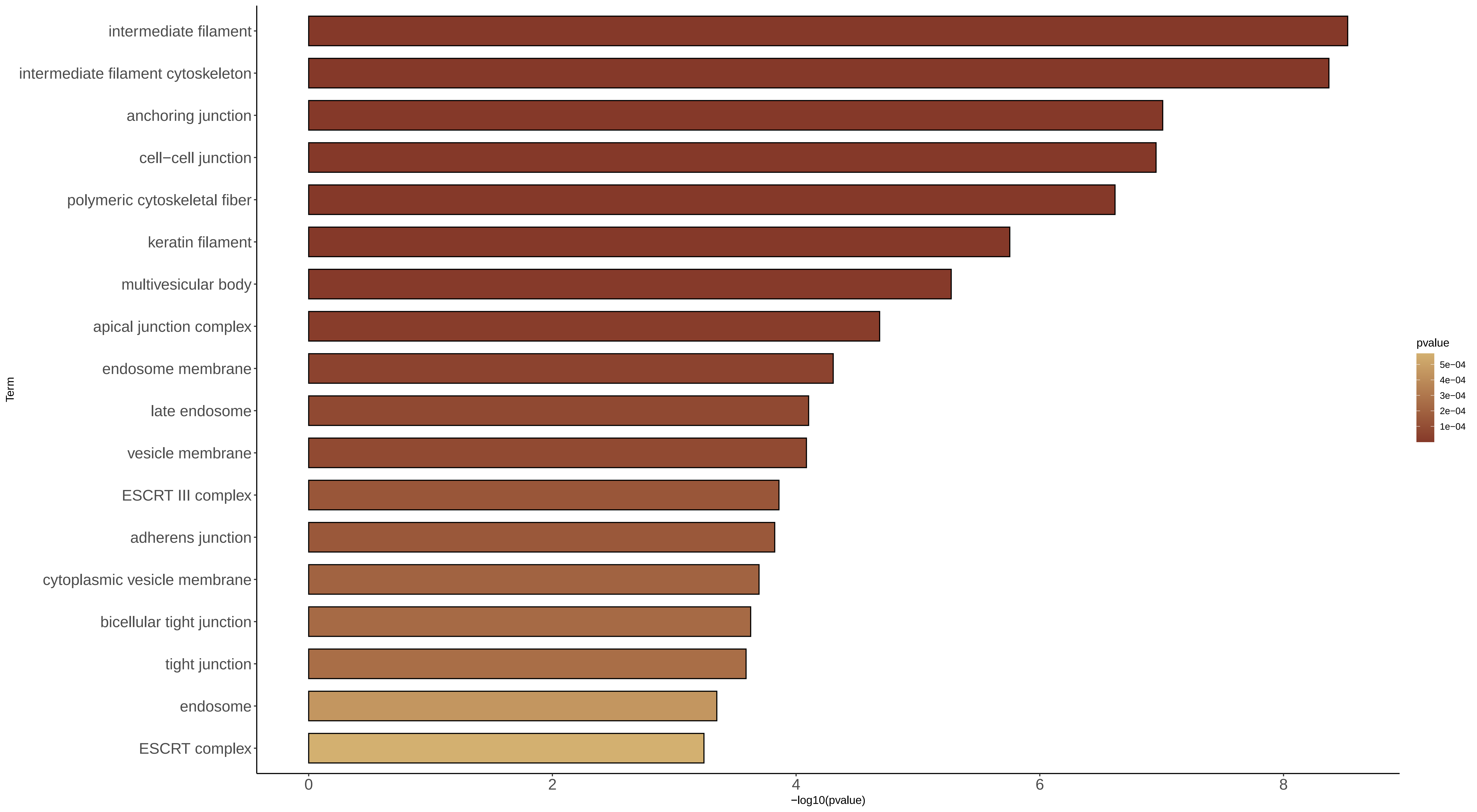

intermediate filament
intermediate filament cytoskeleton
anchoring junction
cell−cell junction
polymeric cytoskeletal fiber
keratin filament
multivesicular body
apical junction complex
endosome membrane
late endosome
vesicle membrane
ESCRT III complex
adherens junction
cytoplasmic vesicle membrane
bicellular tight junction
tight junction
endosome
ESCRT complex
Term
0
2
4
6
8
−log10(pvalue)
pvalue
5e−04
4e−04
3e−04
2e−04
1e−04
